# Aging into tricksters: a qualitative study of women’s positioning and leadership in solar energy communities in Japan

**DOI:** 10.1186/s13705-023-00396-2

**Published:** 2023-05-30

**Authors:** Daniela Lazoroska

**Affiliations:** Aoyama Gakuin University, 4 Chome-4-25 Shibuya, Shibuya City, Tokyo, 150-8366 Japan

**Keywords:** Community energy, Gender, Women, Japan, Situated knowledge, Aging, Trickster

## Abstract

**Background:**

Since the 1960s, women’s social and political engagement in Japan has been closely tied to the roles of mothers and housewives. On the other hand, the country is undergoing considerable demographic changes and has come to be considered an aging society, where an increasing number of women are opting out of marriage and child-rearing. Drawing from qualitative research with women in managerial positions in solar energy communities, this article explores the complex maneuvers informants conducted to fulfill their goal: the expansion of renewable energy in Japan.

**Results:**

The empirical findings point to a new, and underexplored perspective: of aging as a catalyzer for transgressing certain norms and expectations on female behavior in the context of grassroots mobilization. Aging tends to be axiomatically represented as a time of decline, and unidirectional consumption of communal resources. I examine how transitioning from life stages centered on careers and child-rearing offers resources that my informants draw on to sustain their engagement in solar energy communities. I further examine how my informants carefully, and paradoxically, navigate gendered expectations by actively appealing to dominant narratives on women: as attentive communicators attuned to the needs of others.

**Conclusions:**

The article provides suggestions for further diversifying Japan’s community energy landscape, such as (a) increasing awareness of inequalities through open discussion on the topic; (b) gender-inclusive quotas on boards; (c) creating peer-mentoring networks; (d) stimulating a culture of dialogue open to dissensus; (e) shifting the focus away from women needing to make behavioral changes; and (f) not idealizing how much can be achieved without generating discomfort.

## Background

Since the 1960s, women’s social and political engagement in Japan has been closely tied to the roles of mothers and housewives, with motherhood as the centerpiece that catalyzes Japanese women’s collective organization [1–4]. These roles confer moral legitimacy to activities in an unparalleled manner by raising concerns for one’s own or one’s community’s well-being [5]. Contemporary Japan ranks 120th out of 156 nations on the World Economic Forum gender gap rankings [6]. The culprit for this low ranking is women’s limited participation in politics and economic leadership positions. Japan is also undergoing considerable demographic changes and has come to be considered an aging society [7]. By 2036, those aged 65 and over will represent a third of the population, and an increasing number of people are opting out of marriage and having children [7]. Drawing from qualitative research with women in managerial positions in solar energy communities, the empirical findings point to a new, and underexplored, perspective: of aging as a catalyzer for transgressing certain norms and expectations on female behavior in the context of grassroots mobilization. Aging tends to be axiomatically represented as a time of decline [8], and unidirectional consumption of communal resources. This article explores the complex maneuvers informants conducted to morally anchor their actions and fulfill their goal: the expansion of renewable energy in Japan. I examine how my informants carefully navigate gendered expectations by actively appealing to dominant narratives on women: as attentive communicators attuned to the needs of others. I further examine how transitioning from life stages centered on careers, child rearing, or being housewives and mothers if you will, has amounted to a bounty of resources that my informants draw on to sustain their engagement in solar energy communities.

This research contributes by examining new configurations between identity and social action toward a renewable energy transition in Japan. I show how my informants, the female leaders of solar energy communities, aligned their identities and forged inclusive communicative strategies which they saw as enabling them to best perform these roles. My work resonates with the wider body of research examining women’s socio-political engagement in modern Japan and narrows in on renewable energy engagement as a novel empirical and topical focus. As part of the project, 14 qualitative interviews and two field visits have been conducted.

### Theory: the links between the activist, the mother, and the housewife

The process of finding the appropriate literature, contextually and theoretically, was affected by two main conditions. The first condition is that there is limited research on the topic of women’s engagement in energy communities in Japan. Energy community is an umbrella term for supply- and demand-side sustainable energy initiatives, where communities exhibit a high degree of ownership and control, as well as benefit collectively from their outcomes [9]. These initiatives are a relatively new phenomenon. The first initiative in Japan was the Hokkaido Green Fund, which was based on wind energy, and started in 2001 [10]. After the Great East Japan Earthquake, the tsunami, and the Fukushima Daiichi nuclear disaster on March 11, 2011 (hereon 3.11), the number of community-based initiatives have greatly increased. These initiatives were supported by the feed-in tariff system of 2012 [10]. In 2021, estimates indicate the existence of 230 such initiatives [10]. Research that analyses the role and experiences of women in community energy in Japan is at the time of writing (autumn of 2022) confined to a report written by sociologist Furuya Shota [10], a researcher on community energy. Based on a questionnaire survey with 22 respondents in their role as members of the Japan Community Power Association, a nationwide umbrella association, Furuya found that the proportion of women’s participation in such projects and their leadership is low and that actions to increase this ratio are lacking [10]. Furuya writes that 10 organizations have no women, while five organizations have one, and four have two women on the management teams [10]. My qualitative findings confirmed these estimates, with women’s position as leaders in such communities being considered a rarity. Informants tended to recommend each other upon inquiry of further contacts, thus confirming their small numbers. Furuya argued that to achieve greater inclusion in ECs in Japan, quotas could be a way forward [10]. Furuya posits that the Community Power Association has made a positive example, by implementing a 50:50 gender balance for its two co-chair directors and 10 board members [10].

And second, tellingly, it was not obvious if engagement in community energy was perceived as activism, civil society participation, or a job. Drawing on Pierre Bourdieu, David Slater et al. argue that women in Japan engage in ‘classification struggles’ to craft a legitimate position from which to speak in a community and nomenclature, where little space is available for engagement [11]. My informants swiftly obscured my attempts to classify what it is they are doing. Most would say that their focus was on the goal, of combating climate change and providing alternatives to nuclear energy in the face of the triple disaster of 3.11, rather than who they ‘are’ through those activities. This sort of play with roles and identities resonated with the figure of the ‘trickster’. The trickster is a staple in anthropology and related disciplines. The trickster has popularly been described in *Trickster Makes This World* [12], where Hyde examines the figure and its disruptive potential across cultures. There, the trickster is described as a boundary crosser who evades positioning. Nevertheless, for a figure bound to fluidity, Hyde’s trickster is pervasively masculine. In *Sheherezade’s Sisters*, Jurich attempted to pluralize the archetype of the trickster by offering accounts of the role of the female trickster [13]. The female iteration problematizes the trickster’s menacing persona, and of their tricks as self-serving actions [13]. As I will show, this perspective on the trickster has been useful for me to think about informants who worked to balance between fulfilling personal and collective motivations, without being ostracized for performing acts that in some way deviate from gendered social norms. I now present the regional and thematic research that has helped me frame my informants’ narratives that seemingly positioned them as occupying multiple spaces at once. It was in this in-betweenness, or this space outside of themselves, I will argue, that they crafted their agency.

In this research, gender is seen as roles that are socially constructed, made from practices that are repeated and negotiated [14]. This applies to activism too, or as Schieder suggests, ‘women’ and ‘activism’ are not fixed categories but require contextualization [4]. Since the 1960s, Japanese women have been active in various movements. According to Eto Mikiko, women’s movements in Japan, which might differ in their ideological orientation, have all internalized a motherhood tradition [1]. Motherhood is understood as a central value of Japanese women that catalyzes their collective organization [1]. This, Eto argues, reflects Japanese society, which respects women for their motherhood rather than their womanhood [1]. Women’s self-worth is by extension defined through domesticity, marriage, and motherhood [15]. Since the post-war period, women have spearheaded movements associated with consumer rights and the peace movement, and have become visible in their anti-war, anti-nuclear, and environmental activism [4]. According to LeBlanc, these full-time housewives have drawn on their experiences to insist on the value of home-centered perspectives, versus a political economy that prioritizes economic growth over an individual’s quality of life [3]. Ho recognizes that women’s life experiences and identities might not converge with the feminine ideal, but that, nonetheless, there are ideological and institutional pressures to keep women associated with domesticity [15]. Nevertheless, research on gender identity in Japan affirms that informants can find creative ways of expressing their gendered selves, and this asserts their agency in constrained circumstances [3]. Engaging the role of the mother to fuel various kinds of activism and civic activities is not unique to Japan but can be found globally. Drawing on work with activists in Appalachia, for example, Bell and Braun found that women classified their activities as an extension of their traditional feminine responsibilities [5]. This positioning was tied to societal pressure on women to classify familial caretaking as their primary responsibility [5]. As a result, their activities are conferred with moral legitimacy in an unparalleled manner by positioning their claims as based on concerns for their health, or that of their community [5]. This is not to say that maternity is the only source of action for those who identify as women. MacGregor, for example, based on research on activism and politics of care in the US, found that informants drew upon a combination of personal resources to sustain their activism, including spiritual, cultural, political, and intellectual traditions, in addition to maternal ones [16].

The activist identity was not seen as relevant among my informants as a definition of their actions, while from my perspective, this is what they were doing. Schieder defines activism as the engagement by non-institutional actors to bring about social change [4]. In Japan, multiple words are used for activism, such as *Undō -ka* (originating from the 1960s militant protests), *Katsudō-ka* (drawing on the word activity rather than protest, and widely associated with community-based projects), and *Akuteibisuto* [a loan word written in katakana, implying work with NPOs (non-profit organizations)] [11]. However, Slater et al. note, that all these versions are generally seen as too extreme [11]. Thus, while informants used their time to make a positive impact on their localities and wider communities, they always evaded classifying these actions or accepting any identity markers that these actions might entail. This is not uncommon. Ho and LeBlanc have written that Japanese women might engage in social activism and employ collective action, which does negotiate the meaning of gender roles, but they do so in conformity with prevailing gender role expectations [3]. In Slater et al.’s findings, being an activist implies opposition to conservatism. Conservatism is taken to mean that working within given social and political structures is good while being disruptive and putting others in an uncomfortable situation is not [11].

As can be seen, motherhood and the role of the housewife have been pivotal in legitimizing activism and different kinds of socially and politically oriented activity in Japan. Nevertheless, Japan is undergoing considerable demographic changes. It has come to be considered an aging society. By 2036, people aged 65 and over will represent a third of the population [7]. Family structures are also undergoing transformations. In the mid-1990s, only one in 20 women in Japan had never been married by the time they turned 50; in 2015, one in seven women was unmarried [17]. In 2018, the number of babies born was at the lowest level since 1899, when record-keeping began [17]. Thus, I wondered, how these demographic shifts might be reflected in informants’ motivations for investing their time in community energy, and as I will go on to show, what kind of identity claims they make based on their activities. Beyond the activist, the mother, and the housewife, in this article, I also engage with the concept of the ‘other’ [18]. From an anthropological perspective, the other is employed to denote difference, an individual or a group with an identity and access to resources that differ from what is seen to be one’s own. However, following Sarukkai [18], and in resonance with the data gathered, I accentuate that my informants’ other was someone to whom they had a responsibility. They needed to act in a manner that is responsive to the needs or requirements of this other.

## Methods

The research has aimed to gain an understanding of who is considered to be a ‘legitimate’ actor, and what is considered an ‘appropriate’ action in the context of grassroots solar energy organizations in Japan. Women’s experiences have been in focus as this is a group that tends to be excluded, or is a minority, both in the energy industry as well as civil society initiatives focusing on energy production [10]. This article is based on a qualitative approach to research, with ethnographic methods as the main method for data gathering. I have employed semi-structured interviews and field visits, during which, observations could be conducted. I conducted interviews with 14 informants in the spring and summer of 2022. The interviews lasted between 30 min and 3 h. The questionnaire included approximately 25 questions on the informants’ background, how they got into current engagement, their motivations, their history of similar work, their experiences of being women in energy communities, and suggestions for broader recruitment into these organizations. Eight of these interviews were conducted online, through zoom, and six have been conducted in person. Informants could choose if they felt more comfortable with an online or an in-person interview. In general, considering the COVID-19 pandemic, informants preferred to have the interviews online. The two field visits were conducted in the Fukushima and in the Kumamoto Prefecture, where I could see the areas where some solar installations were made, as well as make observations of the informants’ daily life activities and dynamics with their collaborators.

Informants have been recruited through three steps: (1) consulting the member’s list at the Japan Community Power Association (JCPA); (2) consulting previous research on the topic and the organizations mentioned therein [10]; (3) employing the snowball method, by asking respondents to recommend further contacts. As Furuyas’ research indicated, there is a relatively limited number of women who are on the boards of energy communities in Japan [10], thus, the number of people who could be included was also relatively limited, considering that not all contacted persons were able to, or interested in partaking. In terms of the characteristics of the participants, nine of them are female and five are male. The youngest informant is 22 years, and the oldest is 70. Most of the informants are between 55 and 65 years. Most of them have a leadership position in their organizations, or work with administration and public relations, as was the case of the youngest informant. The 14 informants represented 10 different organizations (with several representatives from three of the organizations) throughout Japan.

From a methodological standpoint, there have also been limitations, as my skills in the Japanese language are at a basic level. This certainly posed a barrier to the kind of rapport I could build. I have thus worked with research assistants who have been present for the interviews with persons who do not speak English (10 informants), as well as aided with transcription and translation of these interviews. I have manually analyzed the transcriptions for reoccurring topics and themes that have been presented in the findings. The findings have been triangulated by considering multiple sources, such as the report on gender in energy communities in Japan [10] and previous peer-reviewed findings on women’s political and social engagement [1–4]. The data has been anonymized.

While identifying as a woman might have aided me in establishing contact with the informants, I cannot evaluate this in a manner that might aid this analysis. Indeed, as informants did not speak of their gender and what that might imply for their activities in energy communities, this logic extended to our interactions. However, by expanding the network of informants through their own recommendations, I could support their relationship-building, rather than my own. They agreed to be interviewed not necessarily to aid me but to aid the person who recommended the encounter. This has been made visible based on the many times I have needed to explain where I got their contact information, or how I have gotten to know the person who provided me with it. The times when my own, or my assistant’s search resulted in the contact information, have not yielded any interviews. Hence, my network was built based on the people I found, who agreed to help me based on our mutual interest for energy communities. My knowledge of women’s work in energy communities in Sweden, a topic of a former research project, however, generated what I experience as engagement during the interviews, as they spurred comments and questions that went beyond the questionnaire. Finally, my interest in literature also supported rapport with one particular informant, as we seemed to manage to talk about the topic of this research by talking about novels with similar themes, and about the trajectories of the main characters, rather than of the informant directly.

## Results

### Rooting: situated knowledge

Informants often posited that solar energy was an appealing energy source for women. Their answers aligned with what in social theory is classified as ‘situated knowledge’ [19], implying that knowledge is situated in social landscapes, and gained through gendered bodies. As gathered, women were represented as holding valuable perspectives, that are made accessible through their bodies. It was by practicing the chores that are part of daily life that women are provided with valuable insights about the well-being of their children and family members, about the state of their households and surrounding communities, informants reasoned. Fluctuations in the electric bills, as well as an awareness of the work required to get chores done, provide insights into the role of energy sources in daily life, their price, and power. As one female informant stated:

*Many women are in charge of household finances, so solar energy is a very direct link to the energy that is related to those household finances, so I think it is a very attractive energy source. Because when the sun comes out, it generates electricity, very pleasantly, while you're drying your laundry, it's generating a lot of electricity today! (IF3)*

Male informants confirmed this positioning, that women reproduce everyday life, in its material and social content. This work of care and reproduction extends to a connection with and sensitivity to their environment:

*So, in my opinion, women are more sustainable. In short, women are more serious about their environment when it comes to raising children, aren’t they? Men are not sensitive to such things. (IM5)*

This view on women, as knowing from their embodied position posits solar energy as their preferred choice. Solar energy is available for their sensory consumption, as they are understood to be sensorily attuned beings:

*Women probably think that nuclear power is no longer good for them sensibly (because of Fukushima). Also, wind power is not close to them because the places where it is built are far away. I think that solar power is probably of more interest to women, who judge good or bad based on their senses, and in that way, solar power is closer to them.* (IF4)

This informant’s statement, indicating that women are more critical of nuclear power than men is consistent with research findings from Japan and internationally, which point out that women are less supportive than men of technologies that have considerable health and safety risks [20, 21]. Nevertheless, there is a gap between interest in energy issues, concern for health risks, and attunement to household appliances and expenditures, to investing in solar energy, starting an energy community, or becoming a leading figure therein. I now go on to discuss which skills made informants feel that they were good leaders of such initiatives.

### Shifting: situating (knowledge) in others

When informants discussed the skills that made them apt leaders of energy communities, their communication style was often highlighted:

*I feel that being a woman is merely an advantage. (…) I believe (…) that women may be better at open communication, not just speaking from their own perspective. Perhaps it is because I am a woman, but I don't think about what I can or cannot do so much. I guess people would say that I am not afraid of anything. As long as I can get agreement from the members, I work with those around me on what I need to do, I am not too cautious.* (IF1)

From this perspective, an appropriate leader can be attuned to others’ experiences and positions. This capacity to attune to others seemed to be a common, and gendered, narrative. In June 2022, a colleague, and my research assistant independently of each other enthusiastically shared the news that Kishimoto Satoko was elected as mayor of Suginami Ward in Tokyo. Ms. Kishimoto had a history of activism against water utility privatization [20]. As the ward assembly is dominated by the supporters of her opponent, the Liberal Democratic party and the Komeito, Ms. Kishimoto stated during her press conference: ‘I want to consciously listen to the thoughts of residents who did not vote for me and deepen dialogue and understanding’ [22]. This position that the new mayor seemingly took, of both communication with and working for those that have voted for her, and presumably share her ambitions, but also with those who did not, resounded with what my informants had told me. They considered that women were good at, or at least, had the interest to develop, the capacity to communicate with the ‘other’, with those groups and individuals who had different values and priorities in a way that is respectful and inclusive. Note that this is not a sociolinguistic study that addresses the specificities of the Japanese language and the stylistic stylings of the informants, as this is outside of the scope of my expertise. Sociolinguistic studies of language and gender in Japan have already constituted the thriving research topic of ‘women’s language’, which pays attention to particularities, such as a female lexicon, first-person pronouns, sentence-final particles, honorifics, non-assertive and indirect speech styles, etc. [23]. Relevant to the case at hand, though, strands of this research indicate that the division of labor and roles between men and women, with women’s confinement to domestic space, has stripped them of opportunities for assertive public speech [23]. Findings indicate that some women’s way forward has been to borrow the legitimacy of the ‘other’ whom they are speaking of or speaking for.

During interviews, I also experienced that female informants tried to fully attune to what I am asking about, and what my expectations of an answer on their end could be. They frequently apologized for, in their perspective, not being able to answer in more length to some of the questions part of the interview questionnaire. As well as mentioned, that they hoped that the content they provided me with will fulfill the research aims. A male informant affirmed women’s innateness for communication, as they: ‘have strong power to propagate, power to advertise, power to spread via word of mouth’ (IM5)*.* Female informants affirmed that the capacity to communicate was the key skill that has enabled them to smoothly take on their role as leaders in energy communities. Beyond attentiveness to other people’s needs, appropriate communication is also a capacity to be vulnerable and to find a way of working through vulnerability:

*What I consider to be my problem is that I often say that I don't know too much about electricity. But since I don't know so much, when I talk and negotiate with businesses, I discuss my concern that my ignorance is hindering the business with the members, and they all say that I don't need to know anything about electricity. (…) They say, "If you want to learn, go ahead, but we asked you to take this position because we expect you to connect people and take care of them.”* (IF2)

This informant acknowledges what she finds to be her weakness, or lacking the technical knowledge involved in running a renewable energy association. However, she then affirms that she has something else to offer, the capacity for careful communication (to be read as one instilled with care). The informant draws her legitimacy from the acceptance by the remaining members of the organization’s board, who are male, and who supported the informant in taking on the leadership role. This statement also illustrates that male actors in the renewable energy sector might share prejudice against women as better positioned to perform care and communication roles and might expect that they are delegated such roles.

Learning how communication between multiple and at times polarized parties is enabled by recognizing the position of the other, and learning how to adjust to it, informants reasoned:

*IF4: When I started working in the environmental field, about 30 years ago, there were not so many people aware of the environment, not so much. Now, most people thought of the environment as a very important issue. So when I started working in that field, (…), my older sister said to me, ‘what do you call (it), religion?’ (…). In other words, I was told that you are doing something special, something that special people do, and it is not common at all. It’s not normal.*

*Interviewer: Has your sister changed her mind?*

*IF4: It was good for me because her opinion reminded me (that) what I was doing was not normal so I had to try to explain it to people more. It wasn’t my religion, but it was a really important thing so I tried to explain it more clearly.*

Informants presented their communication skills as being developed within the position, rather than being present *apriori*:

*Well, there are people that I work with, so I always pay attention to how to communicate with them, so I think I have developed communication skills that I can use anywhere, in my daily life, even when talking with my husband.* (IF3)

Being this sort of diplomatic communicator is a valuable skill as there seems to be much at stake:

*(Ms. X) is still not an edgy person among other female business owners; we have seen many more radical women, and in some cases, they cause unnecessary conflicts with private companies and local authorities, which are male-dominated. I feel that such troublesome women are rather hindering the next generation of women’s social advancement.* (IME1)

This informant’s statement might perhaps all too clearly illustrate the dangers for women if they are not as careful and diplomatic in their communication: being perceived as troublemakers. This might ultimately lead to their exclusion, literal, or symbolic. Such consequences, perceived or real, could be a contributing factor to informants minimalizing the focus on themselves as being women in solar energy, but also, not to make differentiations, as I now go on to show, whom their actions are beneficial for.

### Leveling: good for everyone

As informants presented it, their motivation for engaging in solar energy was doing good for everyone, for both women and men, for elderly people and youngsters alike. The motivation was thus a leveler: needing to act in the face of natural disasters and environmental crises involves us all. This perspective aligns with writings on female tricksters, with trickery as a means to explore social alternatives for others [13]. There were three reoccurring strands in the ‘origin of motivation’ narratives, which illustrated the informants’ connection with a wider, and much more complex environmental and social landscape: the disaster of 3.11 as a radical event that stirred their lifeworld, increased knowledge of man-made environmental deterioration, or a series of everyday events that provided some sort of ‘awakening’. As one informant stated, she lived a life completely unrelated to energy production before the disaster (IF7). She was a housewife in another prefecture and worked with arts and crafts. However, the disaster, as she put it, changed her whole world. Faced with such a large-scale calamity, her interests and capacities ceased to matter. What mattered was trying to do something, trying to act to ameliorate the suffering of those affected.

A few informants described events before the disaster, which could be based on receiving new information about environmental deterioration:

*Yes, perhaps, the first time I was interested in solar energy and the environmental thing, was when I was a university student, so almost 30*+ *years ago. Because when I was a university student, the UN first made claims about the climate crisis. I think it was 1989 or something. So (…) I started studying (…) to stop climate change or global warming.* (IF4)

It could also be a series of experiences that reflected the environment and people around them as vulnerable and requiring protection:

*I was engaged in a community related to social issues. I watched a movie regarding the Tibetan problem, and it stroke a chord with me, so I held a meeting for the movie. When I encounter something that touches my heart, then I want to spread it to people, people who are in my local community.* (IF1)

As this informant’s statement illustrates, she had an experience that ‘touched her heart’, something that made her see a new set of connections with the world, and this urged her to act.

Not only was solar energy production good for everyone but engaging in energy communities, I was told, was also possible for everyone, despite their gender or other identity traits and social roles. Difficulties were rendered as an individual matter:

*I don’t think that I’m having a hard time because I’m a woman, and I take the lack of necessary skills as a personal problem.* (IF1)

Positioning difficulties with participating in energy communities as an individual’s responsibility absolves the organization from examining the obstacles that the organizational form and structure might pose. Neither does it address the circumstances that different groups experience. Individuals are made visible in their vulnerability, but groups remain protected. My informants are now leaders (chairperson of the board) of these organizations, they have made it to the other side. Thus, they take on the responsibility for any struggles themselves but leave their energy communities intact, and deflect criticism from them.

Avoiding the discussion that participation in energy communities can be harder for some groups rather than others, can also be seen as a conscious maneuver in an environment hostile to working with group-based interests. For example, on the question of women’s limited political participation in Japan, Professor Taniguchi Mayumi stated in a documentary that a lot of female politicians are not advocating for women’s rights, as party politics is male-dominated, and as it is easier to get through an election as a woman if one avoids topics regarding women completely [24]. Success as a woman in a masculine environment can require a certain level of disavowal of one’s identity as a woman and of gender-based inequality as a matter of concern. I now go on to discuss how my informants’ roles in leadership positions in energy communities might be aided by their life stages and the privileges that aging can confer.

### Aging into tricksters

In the *Emissary* [25], by Tawada Yoko, a dystopian novel, the reader encounters a post-disaster Japan, where for undisclosed reasons, the elderly are left with potentially eternal life and exuberant energy. The young people, on the other hand, are frail, wise beyond their years, and in need of caretaking in an environment that is increasingly dangerous for their survival. Gender is not fixed, and characters go through several transitions throughout their lifetimes. These literary narratives, about the well-resourced elderly needing to do what they can to provide a better future for coming generations, are also present in the accounts of my informants. I tie their narratives about aging to the literature on tricksters [13], as aging seems to provide my informants’ access to spaces and roles that have predominantly been associated with men. Informants were not bound to justify their actions as being based on traditional gender roles, such as motherhood or being housewives. They could draw on different roles they have had throughout their lives, because age has exposed them to a variety of experiences.

Most of my informants were aged between 55 and 65 years. Only three were in their early 20 s and 40 s, respectively. Not all of them had children. Those who did have children had teenagers or young adults. As I wrote at the outset of this article, women’s social and political engagement in Japan has been closely tied to the roles of mothers and housewives, with these roles as moral legitimizers for women’s actions. Despite there being fewer who can or wish to identify with these roles, they are in no way a remnant of the past, and they still leverage many women’s public actions. The informant who is in her early forties told me that she does not identify as an activist. However, she understands that people like me (read: researchers) would think of her as one. She explained that she is a mother and a farmer. As our conversation went on, she told me that even after nearly two decades of living in the area, she is still considered an outsider by her neighbors. However, reminding them that she has raised her children in the area provided a certain level of insider-ism. These experiences, of long-term residence and becoming a parent in the area, she added, also leverage her negotiations with the local government for investing in more renewable solutions. They make this informant seem more credible for the local government actors, the informant reasoned, as she had over time accumulated life experiences and a social network that they might share. This informant explained that as a woman she would be more trusted in certain respects, but not others, such as business and economic plans. To an extent, her identity as a university-educated woman from a large city might be in the way of making claims. This side of her identity, although it might be informing her positions, is thus occluded in her interactions with the local government. The informant’s statement illustrates her belief that different aspects of her identity will provide her with leverage with different groups. She perceived researchers as willing to classify her as an activist, or her local government officials and neighbors as a mother. While such acts of code, or identity switching, are indeed skillful, they also affirm certain prejudice, of, for example, women’s authority being derived through motherhood.

Women’s employment in Japan has tended to be shaped as an M curve, implying that women leave the labor force in their 30 s and 40 s, to perform care labor for families, returning when children are aged 15 or older [26]. The informants that had passed their fifties did not state that they were in the position of needing to care for a family member. They had either quit ‘regular’ jobs they had before or could make their engagement in the organizations a paying position. In general, they seemed to have reached relative material stability. They thus had different kinds of resources at their disposal which they could draw upon, even in terms of narratives about themselves and roles they chose to highlight. For example, the following informant, who has a natural science university degree, quit her company job in the late 1990s, at age 40 to work for organizations with pro-environmental goals. At the organization she is currently at, she is a sort of jack-of-all-trades:

*We need to do a lot of technical things, to build power plants, but other employees specialize in that, and I have a lot of different jobs in the company, such as monitoring the power plants to make sure they are generating electricity properly and monitoring the income from the sale of the electricity. I also do administrative work, which I think is useful. And each power station has its own management company. I also play that role, and it is necessary to have a smooth relationship with them, so I do my best in that area.* (IF9)

My informants have come to a life stage, where active care for dependents was not as actively exercised or expected of them as it might have been in earlier life stages. They seemed to be able to opt out of the daily care for the home and housework. As one informant put it: ‘my life has become work-centered, so the housework got messed up. Meals and cleaning are not done properly’ (IF1). Their skills and authority were derived from a different kind of situatedness. They were not based on the gendered and embodied experiences that they claimed to be central to other women’s interest in solar energy. Rather, they were situated in their social networks and had an acute awareness of social cues.

While there was an acknowledgment that the relative privileges that they have gotten as they have aged, informants still expressed a concern that there were not enough young people on their boards. As one informant put it:

*(the other woman on the board and I) always talk about how we need young people to inherit what we are doing. People who are involved in (this organization), including me, they're all over 50 years old. The owners are very old, so we have been talking about needing young staff.* (IF4)

Informants did nevertheless have reservations about how many younger people they could get on their boards, as these people would lack the time, and would only be interested if the remuneration was competitive. In line with Slater et al., we can see that they engaged in the aforementioned ‘classification struggles’, by explaining that their work with these organizations was their hobby, or that there was no differentiation in their lives between different activities. This level of engagement could not be expected by someone who is actively building a family and a career, I was told.

## Discussion

I began this article by presenting how women’s public social engagement in Japan has been tied to the roles of housewives and mothers. Tapping into these roles can be a way of conferring moral legitimacy to one’s actions and visions. Nevertheless, in light of Japan’s changing demographic landscape, the connections between social action and identity are bound to become reconfigured. As I have illustrated from the findings, informants addressed the roles of mothers and housewives by projecting them onto other women. The concept of ‘transversal dialogues’ can shed some light on this approach. Nina Lykke explains that transversal dialoguing requires being able to shift between ‘rooting’ (situating own stakes as situated knowledge), and ‘shifting’ (trying to imagine what it takes to inhabit the situated perspective of the other) [27]. Interestingly, female informants did tap into the links between women’s interest in solar energy being situated in their daily work in the household. However, they did not acknowledge this presumed connection as enabling their own roles. This position legitimizes a connection between women and energy and justifies that it would be a matter of concern for this group, but it does not explain their way toward leadership. Their resourcefulness as leaders of energy communities was based on inclusive communication strategies, they told me. As Jurich aptly notes, women have less access to spaces that men can easily traverse [13]. Thus, they need to ‘talk their way’ into power and different positions [13]. Some of the tricks that female tricksters can rely on, to persuade, it is argued, are diction and eloquence [13]. The communicative strategy my informants employed can also be a way to ward off any suspicion that the actions are coming from, or are aimed specifically toward women’s concerns. As they cared to remind me, energy communities were good for everyone. They are thus ‘unrooted’ from womanhood and its links to embodiment and care labor in the home. They highlighted the skill of being able to tap into the perspective of others, or ‘shifting’. Rooted in something that ‘other’ women are, and my informant’s way of rooting was to shift at the outset, or root in the other, as it were. These tricksters narrated their way between identities and roles. Careful communication is in this respect a creative act in which the one performing it can manage the change they desire [13].

This movement seemed to be enabled by aging, and by being able to try out multiple roles: as students, mothers, housewives, employees in different sectors, et cetera. The literature that addresses aging and ageism in relation to a just energy transition is a budding field [28]. These perspectives highlight the discrimination of older people in the context of the energy transition, particularly when coupled with misogyny, racism, ableism, homophobia, classism, and other biases [28]. Considering that most of the subjects in the study have passed their 50 s, aging seemed to confer many material and experiential resources upon them. They did not present needing to perform active care labor in their daily lives, thus, they could choose to confer their attention to a cause they saw fit, in the case at hand, being community energy. Nevertheless, the subjects included in this study have relatively privileged class positions, as could be derived from their educational and employment history. As a possible venue for further investigation, at a time when an on-site meeting does not pose a health risk, I propose an observation-based study that takes into consideration these subjects’ interactions with their collaborators from an intersectional perspective, sensitive to how gender, age, class, and other identity markers might affect action and agency.

Nevertheless, I do not wish to present flexibility in positioning and attentive communication as a normative practice. Rather, by drawing on research on gender identities in Japan, and gathering data, I hope to show that being socialized as a woman in Japan can set the stage for the communication style described herein. As women are subordinated in patriarchal systems, communication skills that can accommodate the position of the other can be seen as both a tool for survival, as well as for asserting agency. Herein lies the conundrum, considering conformity to normative gender roles as a mode of asserting agency. According to Kimura, when women engage with social issues in Japan, they are expected to perform a cheerful, friendly, and unthreatening femininity [2]. LeBlanc confirms these findings by writing that researchers looking at gender identity in Japan find their informants asserting agency in constrained circumstances by making creative use of gendered self-presentation strategies. Both men and women may work to take ownership of their lives by taking on gendered voices they know are not their own [3]. This is a paradoxical use of constraint in resistance to constraint, according to LeBlanc [3]. This could be exemplified by, say, a rural woman who adopts Tokyo women’s more feminized language, or a Tokyo woman who uses her housewife identity to assert herself as a pro-environment candidate for elected office [3]. What have my informants been able to achieve by rooting in the other and shifting? They have been able to carve out a space of action. They have been able to maintain relationships and build alliances by being empathetic listeners.

Informants worked with this tenuous balancing act, of highlighting certain aspects of their identities while occluding others. Therefore, why would they claim to not notice gender, that their gender was never an obstacle? As one informant’s quote indicates in the section *Shifting: Situating (knowledge) in others*, standing out, and being what is seen as conflictual is not rewarded. One is expected to present themselves as agreeable, and capable of working on and supporting relationships, which may have aided them in affirming their positions and building alliances. In a way, however, while evading gendering, my informants do take on a gendered role, by becoming responsible for emotional care labor, and the maintenance of relationships, a task traditionally ascribed to women. Perhaps as a woman, a minority in the sphere, one already stands out, so then to avoid friction, one tries to hide behind the claim of being gender-free and one’s actions having collective value. The trick a trickster plays can benefit the individual performing it. It can claim freedoms for the trickster from the impositions of society and culture [13]. However, it can also aim to bring benefits to the community [13]. The trickster sometimes needs to distance themselves from the self to challenge self-centeredness [13]. In previous research from Sweden, our research team also encountered similar claims [29], that renewable energy is for everyone. Indeed, renewable energy should be accessible to everyone, as a product to consume and produce. However, what does this claim to collective value also do? I argue that it can also, perhaps inadvertently, occlude power relationships, and allow for the goals of environmental sustainability to tramp upon the social sustainability of the organization itself. Those in leadership, both from the aforementioned research in Sweden, and Japan were the usual suspects, middle-aged, middle-class men. This group has more resources available (such as money, time, etc.) that they can use to invest in such initiatives [29, 30]. I would also argue that women or minorities in general in energy communities might be supported by having a network of peers, where they could balance collective pursuits while acknowledging their particular social positions and experiences. Research findings from the USA and Canada’s renewable energy sector affirm the positive role of peer mentoring for professional development [31]. Such networks might stimulate the diversification of similar organizations in Japan, which at the time seemed to have difficulties including women and other groups from the general population of Japan.

On the other hand, the importance of combating climate change also erases the differences between the hands that need to be on deck. We are all equal in the face of environmental degradation, although how we experience it runs along class, race, and gender lines [32]. Claim to universality can thus be seen as a communicative action aiming to be inclusive and mobilize across the board. Nevertheless, considering the limited number of women in managerial posts, one can consider that these organizations have unequal power structures and that grassroots renewable energy production in Japan suffers from a lack of diversity in its ranks.

## Conclusions

In this article, I have shown how women’s social engagement has moved through its entanglements with mother and housewife roles as sources of legitimacy, toward tricksters evading positioning. This is enabled by a process of careful selection, presentation, and occlusion of their life experiences, motivations, and roles. While this is employed as a strategy to be empathetic communicators and ward off any suspicion that the actions are bound to women’s concerns, they are not contributing toward greater inclusivity in energy-related grassroots initiatives. Indeed, this is a side-effect, as evasion occludes the inequality in these grassroots initiatives. This research has been primarily based on interview data, which has had its strengths in giving the informants space to voice their own concerns. On the other hand, it is also the limitation of the article, as more observation-based data would provide insights into how these negotiations are made, and what lies beyond the interviewee’s self-reflection on how gender and other identity markers enable or constrain action. While I have in this article showed how aging has bestowed the informants with resources (such as work experience and time available) that they can use in their roles as leaders of energy-focused organizations, one must be attentive to the possible discrimination that subjects could experience because of ageism, for example.

The following proposal for greater inclusion and diversity in community energy is made based on my findings. I argue that there (a) needs to be a collective acknowledgment that women are a minority in the organizations and that further modes of action need to be agreed upon. Considering the very limited participation of women in managerial positions, (b) quotas could be a way of effectively increasing equality and inclusion, following the positive example of the Community Power Association. Furthermore, (c) peer networks of women in renewable energy in Japan could also be a way of attracting and retaining more staff/volunteers, as well as supporting innovation in such initiatives. I would add that it is also essential to (d) provide an atmosphere welcoming of dissensus and diversity within these organizations. In grassroots initiatives, as well as across societal groupings, women should not expect less than safe forums, where they can openly communicate and not be penalized or excluded for thinking differently. Women’s participation in community energy, or lack thereof, should (e) not be reduced to behavioral change discussions, expecting women to change their behavior, to make more time, to be more amenable, or more assertive to be able to partake, or be leaders. One needs to examine the conditions of the organizational form, what kinds of requirements and expectations for participation at various levels there are, whom they open for, and whom they exclude. Finally, (f) one cannot idealize how much can be achieved without generating discomfort. Working toward diversity and inclusion requires it.

## Data Availability

The data contains personal information and hence cannot be made public.
